# Supplementary material for: Ethylene-triggered subcellular trafficking of CTR1 enhances the response to ethylene gas
Source: Nat Commun. 2023 Jan 23;14:365. doi: 10.1038/s41467-023-35975-6 (PMC9870993; doi:10.1038/s41467-023-35975-6)
Supplement: Supplementary file 1 — Supplementary information [file 41467_2023_35975_MOESM1_ESM.docx]

Ethylene-triggered subcellular trafficking of CTR1 enhances the response to ethylene gas.

Hye Lin Park^1,2,†^, Dong Hye Seo^1,2,¥†^, Han Yong Lee^1,2,$,†^, Arkadipta Bakshi^3#^ , Chanung Park^1,2^, Yuan-Chi Chien^1,2^, Joseph J. Kieber^4^, Brad M. Binder^3^, and Gyeong Mee Yoon^1,2,*^.

*To whom correspondence should be addressed. E-mail: yoong@purdue.edu

**This PDF file includes:**

Supplementary Figures 1-21

Supplementary Table 1-3


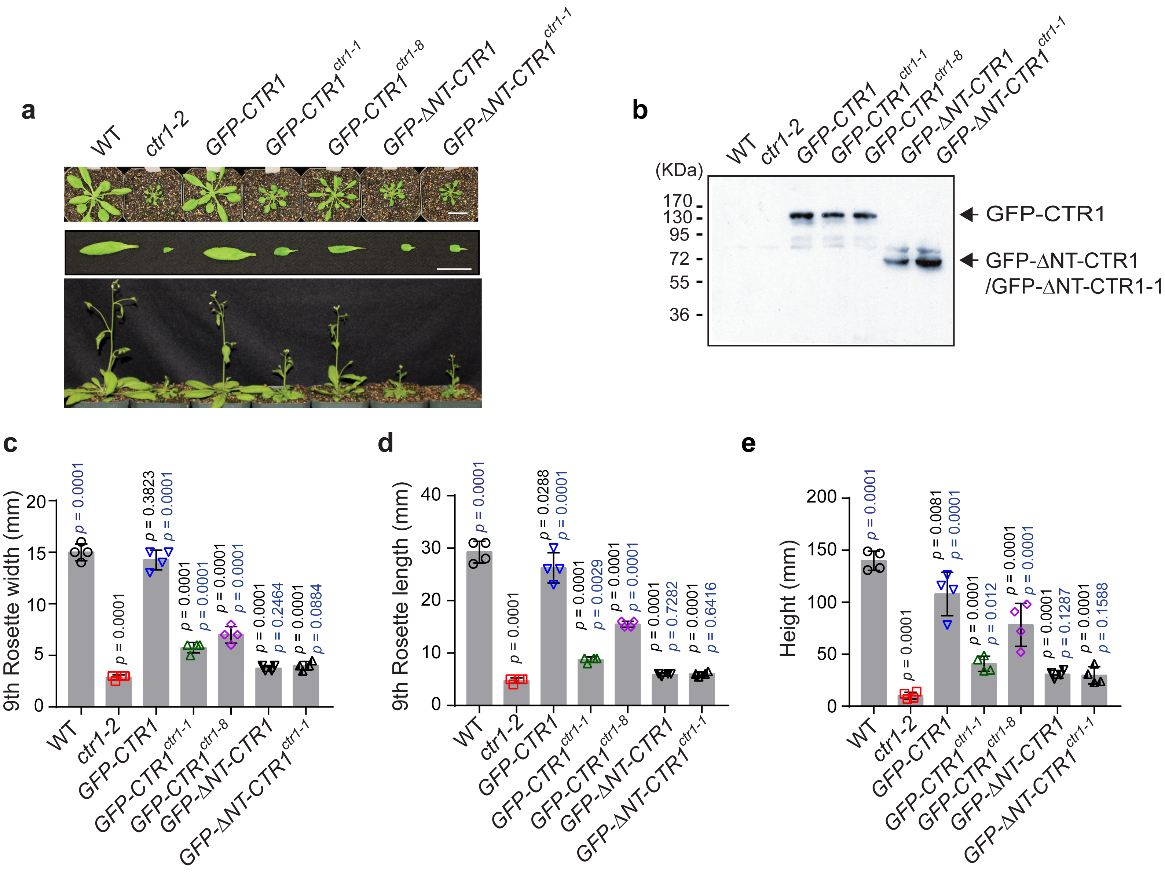


Supplementary Figure 1. Rescue of *ctr1-2* by *CTR1* transgenes in light.

a, Representative 2-week-old (top two rows) or 4-week-old (bottom row) light-grown wild-type and transgenic lines expressing various genomic *CTR1* transgenes from the native promoter in the *ctr1-2* background. Scale bar, 20 mm. b, Western blot analysis of GFP-fused CTR1 proteins expressed in seedlings in (a). c-d, Quantification of the width (c) and length (d) of the 9^th^ rosette leaves of 2-week-old plants in (a). Data represent the means and SD (*n*=4). e, Quantification of the height of 4-week-old wild-type and transgenic plants in (a). Significance was determined by one-way ANOVA, Dunnett's multiple comparisons test compared to WT (black) and *ctr1-2* (blue). Error bars, SD (*n*=4 plants).

**
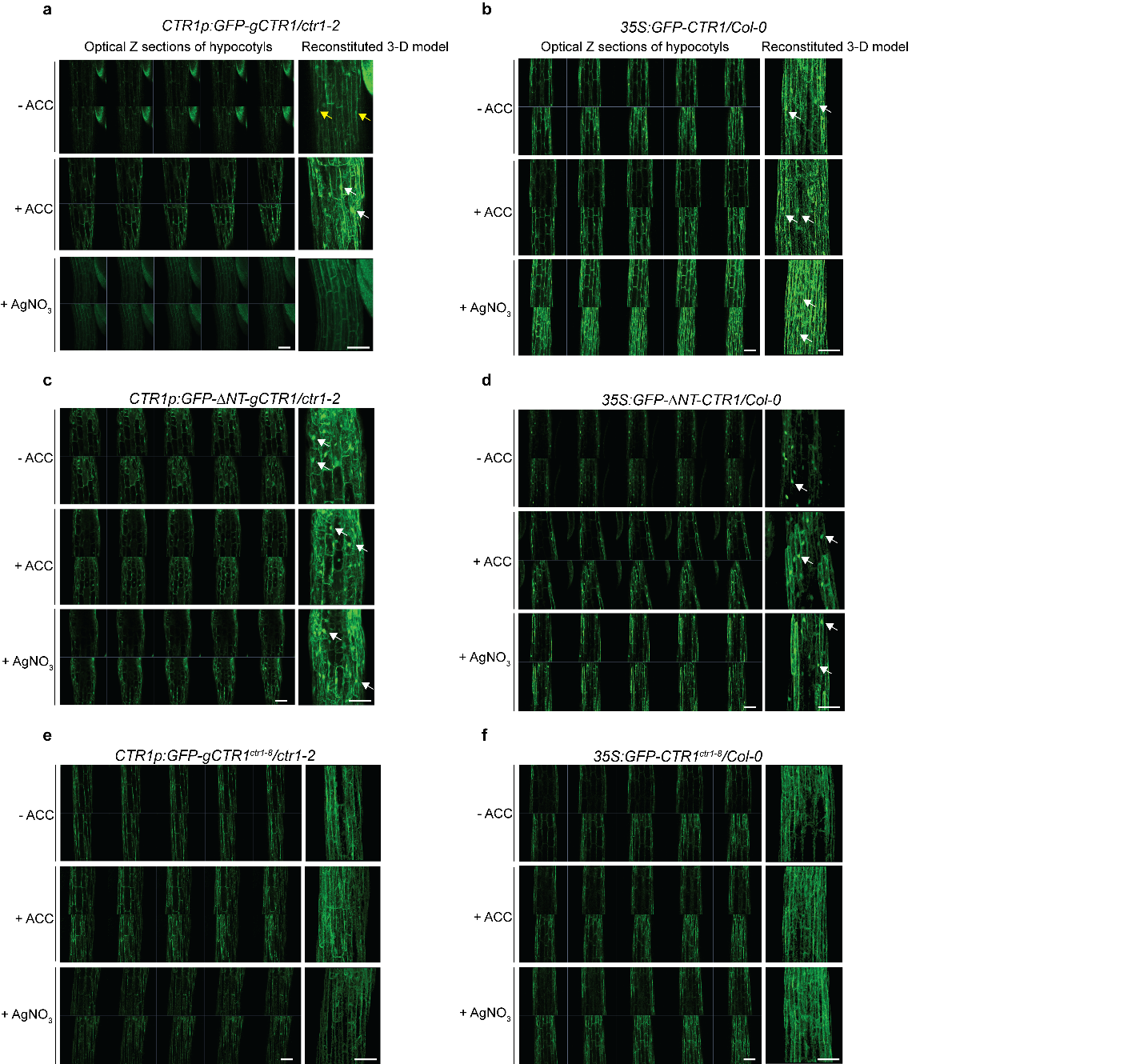
**

**Supplementary Figure 2. Z-stack images of seedlings expressing WT CTR1 or CTR1 variants from the 35S CaMV or its native CTR1 promoter.** Seedlings were grown on MS medium with 100 µM AgNO_3_ in the dark or 3-d-old etiolated seedlings grown on MS without AgNO_3_ were treated with or without 200 µM ACC for 2 h before imaging. The areas below the hook and above the elongation zone of the hypocotyls of dark-grown seedlings were imaged. Each montage contains 10 of 2 µm optical sections. White arrows indicate the nuclear-localized CTR1. Yellow arrows indicate the basal levels of nuclear-localized CTR1 in etiolated seedlings without any treatment. Scale bars, 50 µm.

**
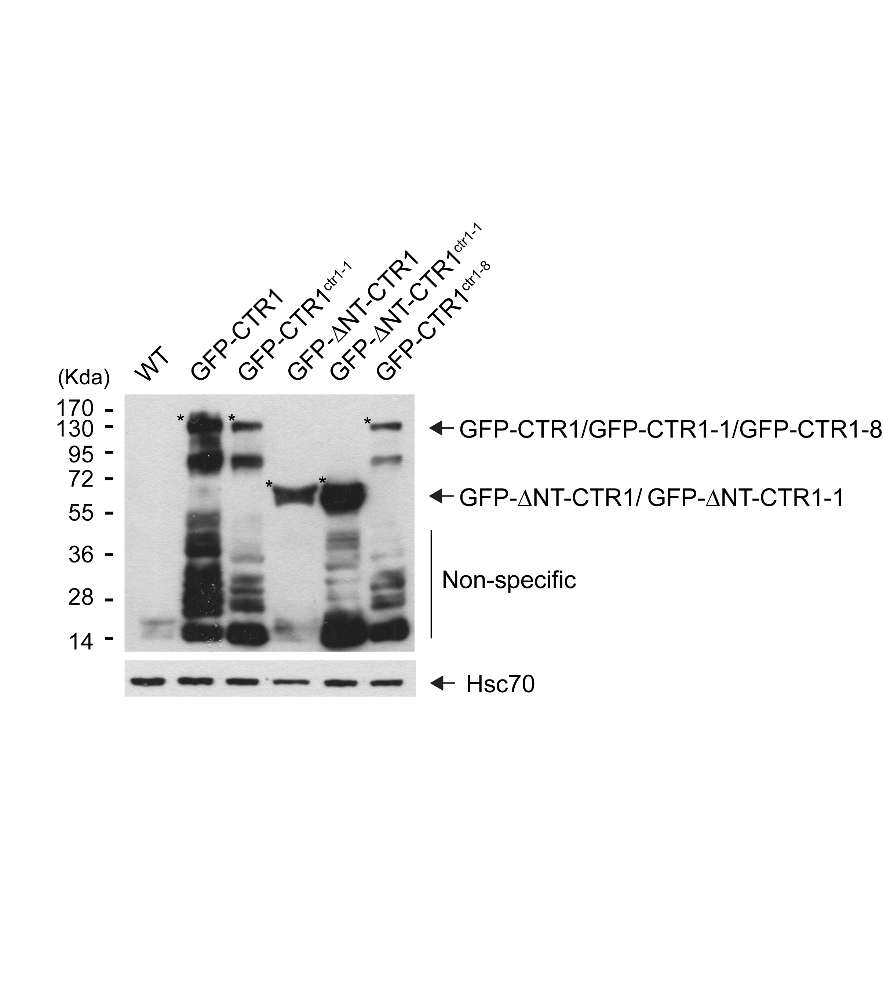
**

**Supplementary Figure 3. Western blotting analysis of GFP-fused CTR1 in 35S promoter-driven overexpression lines.** The total protein extracts of 3-d-old dark-grown seedlings were subjected to immunoblotting analysis with anti-GFP and anti-Hsc70. * indicates the corresponding protein bands. The results were reproducible in three independent experiments.


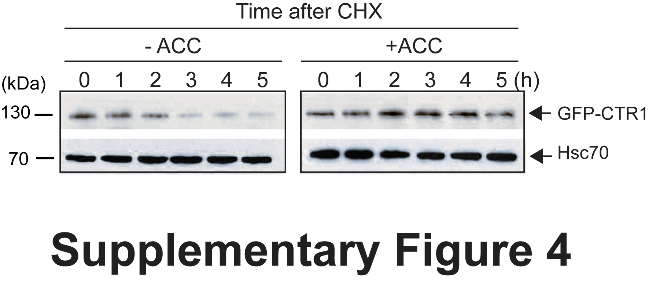


**Supplementary Figure 4. Protein degradation analysis of CTR1 with or without ACC treatment.** The etiolated wild-type seedlings were treated with or without ACC (200 µM), followed by cycloheximide (CHX) treatment. Seedlings were harvested at different times after CHX treatment, and total protein extracts were analyzed by immunoblotting with anti-GFP and anti-Hsc70 antibodies. The results were reproducible in three independent experiments.


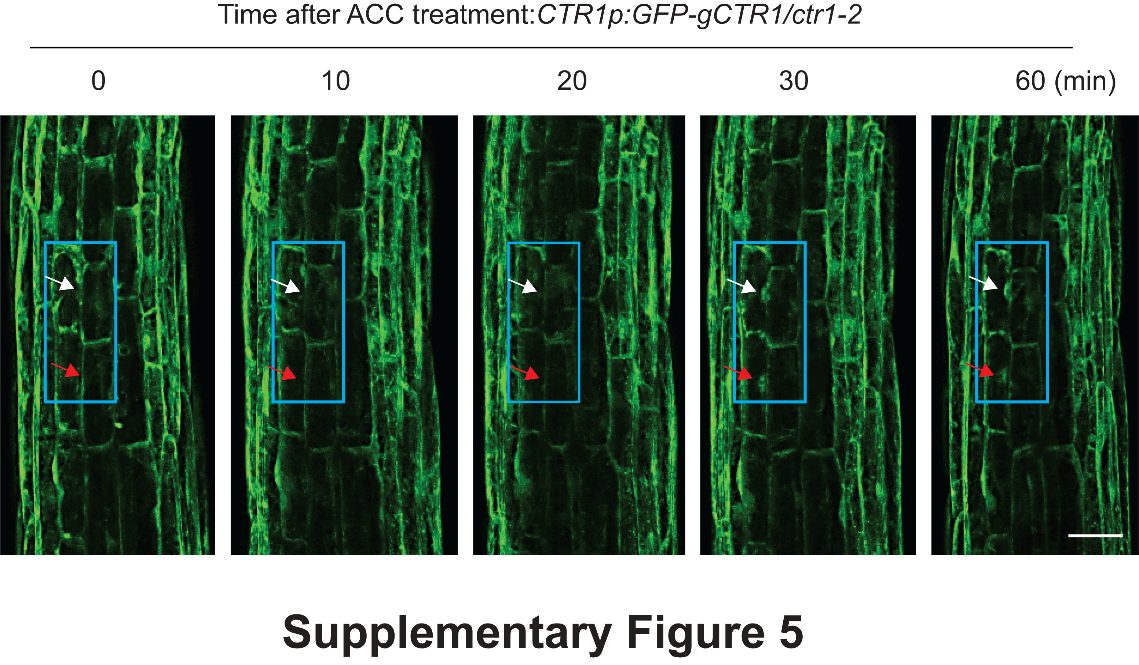


**Supplementary Figure 5. CTR1 appears to translocate into the nucleus within 30 min after ACC treatment.** Time-lapse Z-stack image series of hypocotyl cells expressing GFP-CTR1 in 3-d-old etiolated seedlings after exposure to 200 µM ACC, visualized by confocal microscopy. Arrows track specific cell nuclei, showing the accumulation of GFP-CTR1 in response to ACC. Scale bar, 50 µm. The presented images at each time point are merged Z-stack images that combine 10 of successive Z-stack images with 2 µm optical sections.


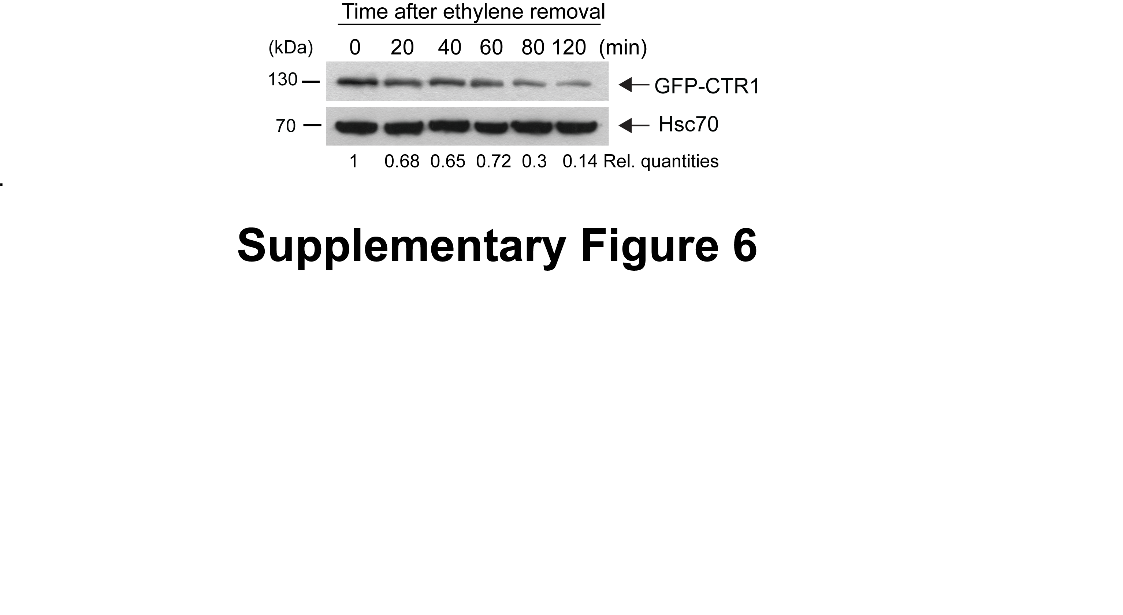


**Supplementary Figure 6. The steady-state levels of CTR1 gradually decrease after ethylene removal.** Seedlings were pre-treated with 10 ppm ethylene for 2 h and harvested at the different time intervals indicated. Total protein extracts were then subsequently analyzed by immunoblotting using anti-GFP and anti-Hsc70 antibodies. The results were reproducible in three independent experiments.


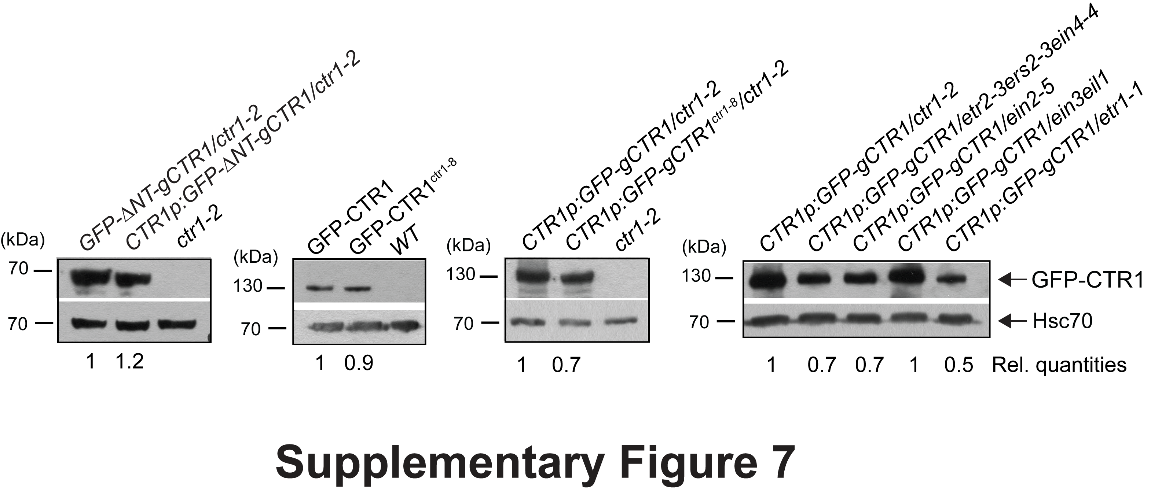


**Supplementary Figure 7. The expression levels of WT CTR1 and CTR1 variants in seedlings.** The total protein extracts of 3-d-old seedlings expressing WT CTR1 and various CTR1 variants from the 35S CaMV promoter or the native CTR1 promoter used in Supplementary Fig. 2 and this study were analyzed by immunoblotting using anti-GFP and anti-Hsc70 antibodies. Rel. quantities represent the ratio of the intensity of the GFP-CTR1 bands to Hsc70 band signals, and these values are expressed relative to the intensity of CTR1/Hsc70 in the first sample of each blot, which was set to 1. The results were reproducible in three independent experiments.

**
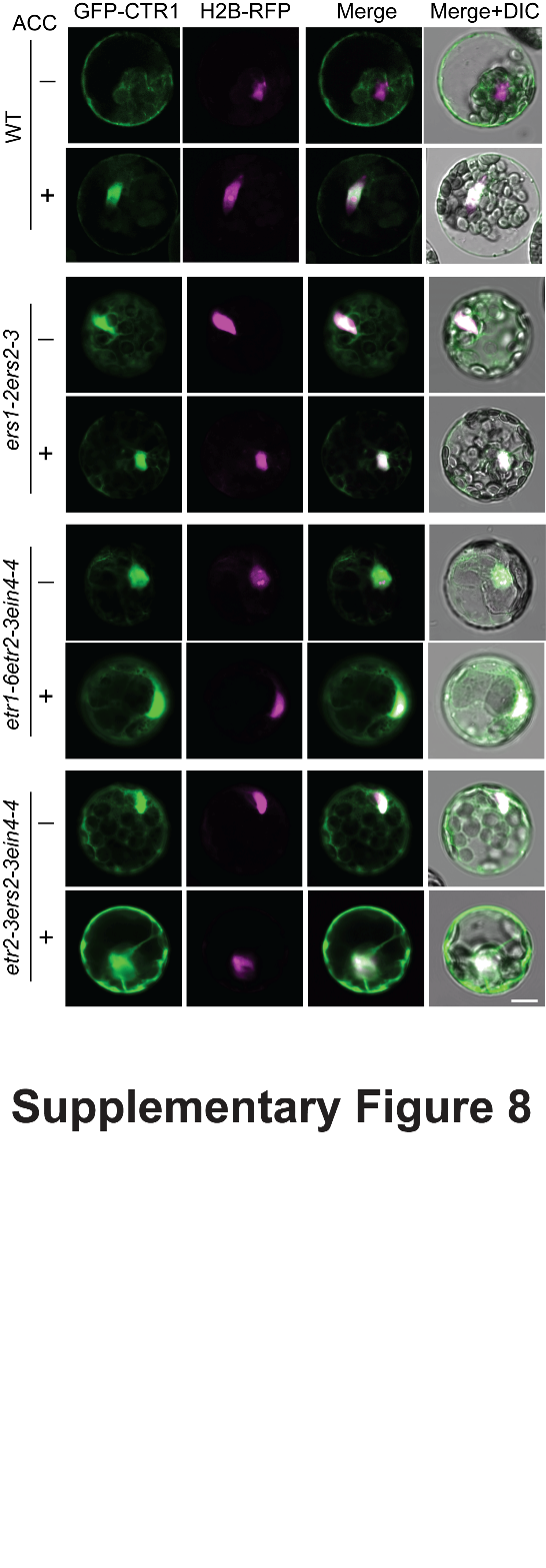
**

**Supplementary Figure 8. Subcellular localization of CTR1 in various ethylene receptor mutants.** *Arabidopsis* mesophyll protoplasts from indicated high order ethylene receptor mutants were transfected with a *GFP-CTR1* plasmid, and the subcellular localization of GFP-CTR1 was observed by confocal microscopy. H2B-RFP is a nuclear marker protein. Scale bar, 10 µm. The imaging was performed more than three times with similar results.


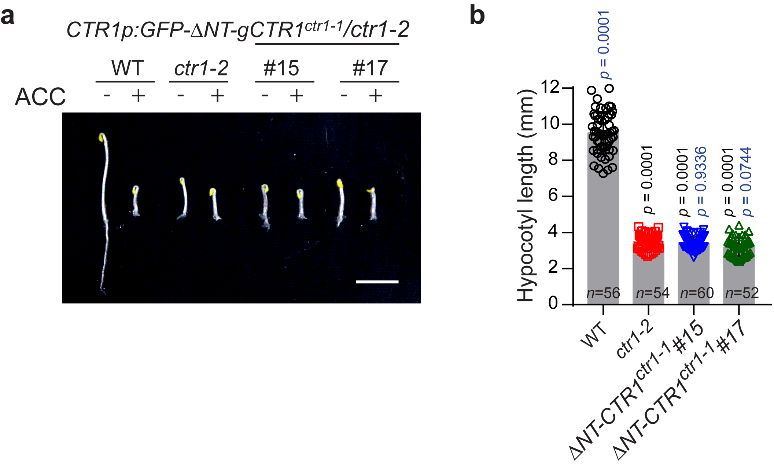


Supplementary Figure 9. The transgene encoding the CTR1 kinase domain without kinase activity does not rescue *ctr1-2*. a, Representative images of etiolated wild-type and seedlings expressing the CTR1 kinase domain only with *ctr1-1* mutation grown on MS medium with or without 10 μM ACC. Scale bar, 5 mm. b, Quantification of hypocotyl lengths of seedlings in (a) without ACC treatment. *P*-values were determined by one-way ANOVA, Dunnett's multiple comparisons test compared to WT (black) and *ctr1-2* (blue). Error bars, SD.


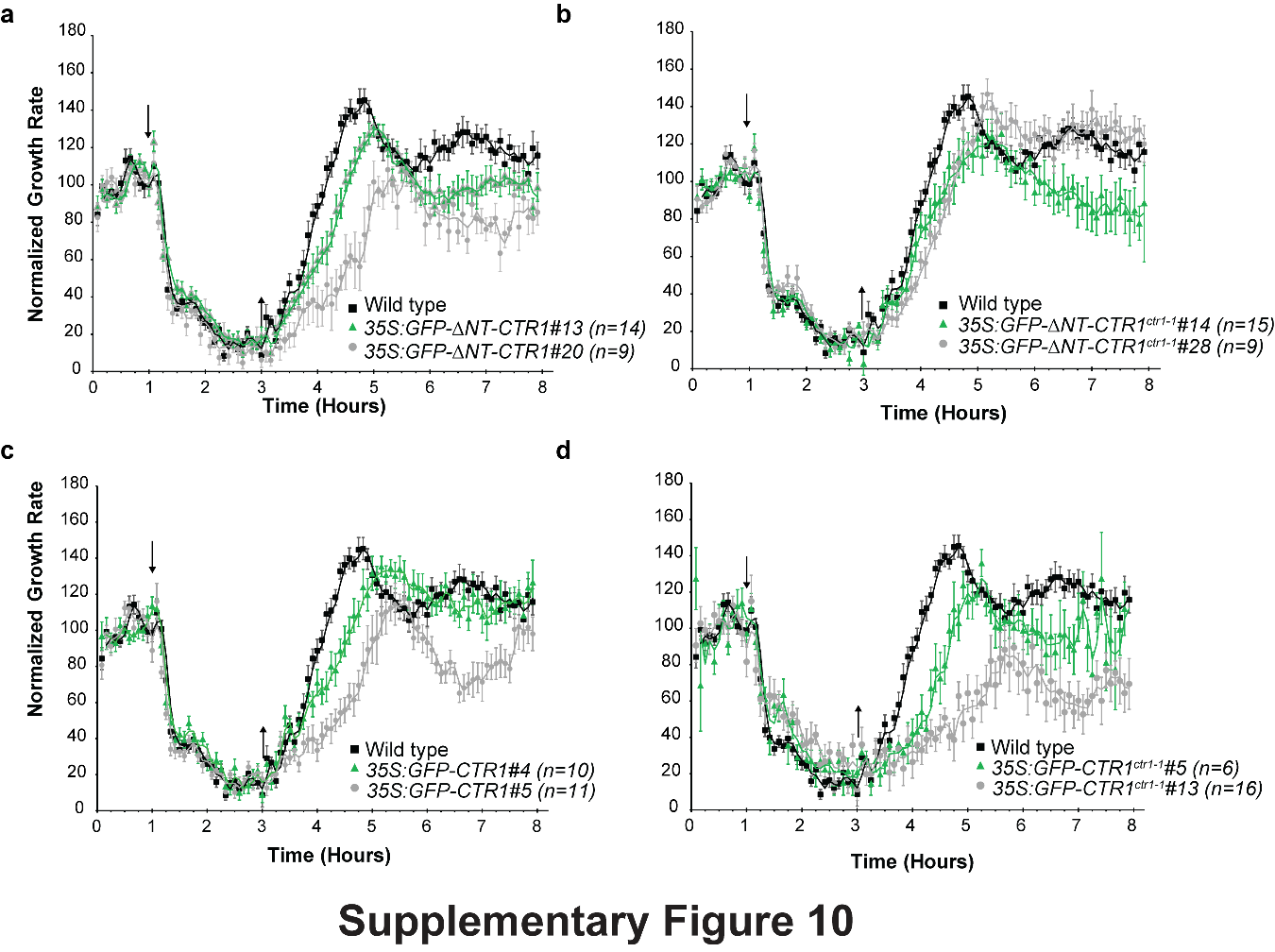
**Supplementary Figure 10. Nuclear-localized CTR1 delays the growth recovery of hypocotyls after ethylene removal.** The hypocotyl growth rate in response to ethylene was recorded for 1 h in air, followed by 2 h exposure to 10 ppm ethylene and then 5 h recovery in air. Ethylene was introduced 1 h after measurements were initiated (down arrow) and then removed 2 h later (up arrow). The responses of wild-type seedlings are shown in each graph. **a**, *35S:GFP-ΔNT-CTR1*. **b**. *35:GFP-ΔNT-CTR1^ctr1-1^*. **c**, *35S:GFP-CTR1*. **d** *35:GFP-CTR1^ctr1-1^*. Two independent lines of each genotype were used for analysis. Green lines represent the overexpression lines presented in Fig. 4 and grey lines represent an additional independent line of corresponding 35S lines. The data were normalized to the growth rate in air before treatment with ethylene. Error bars, SE. The experiments were repeated at least twice and generated similar results.

**
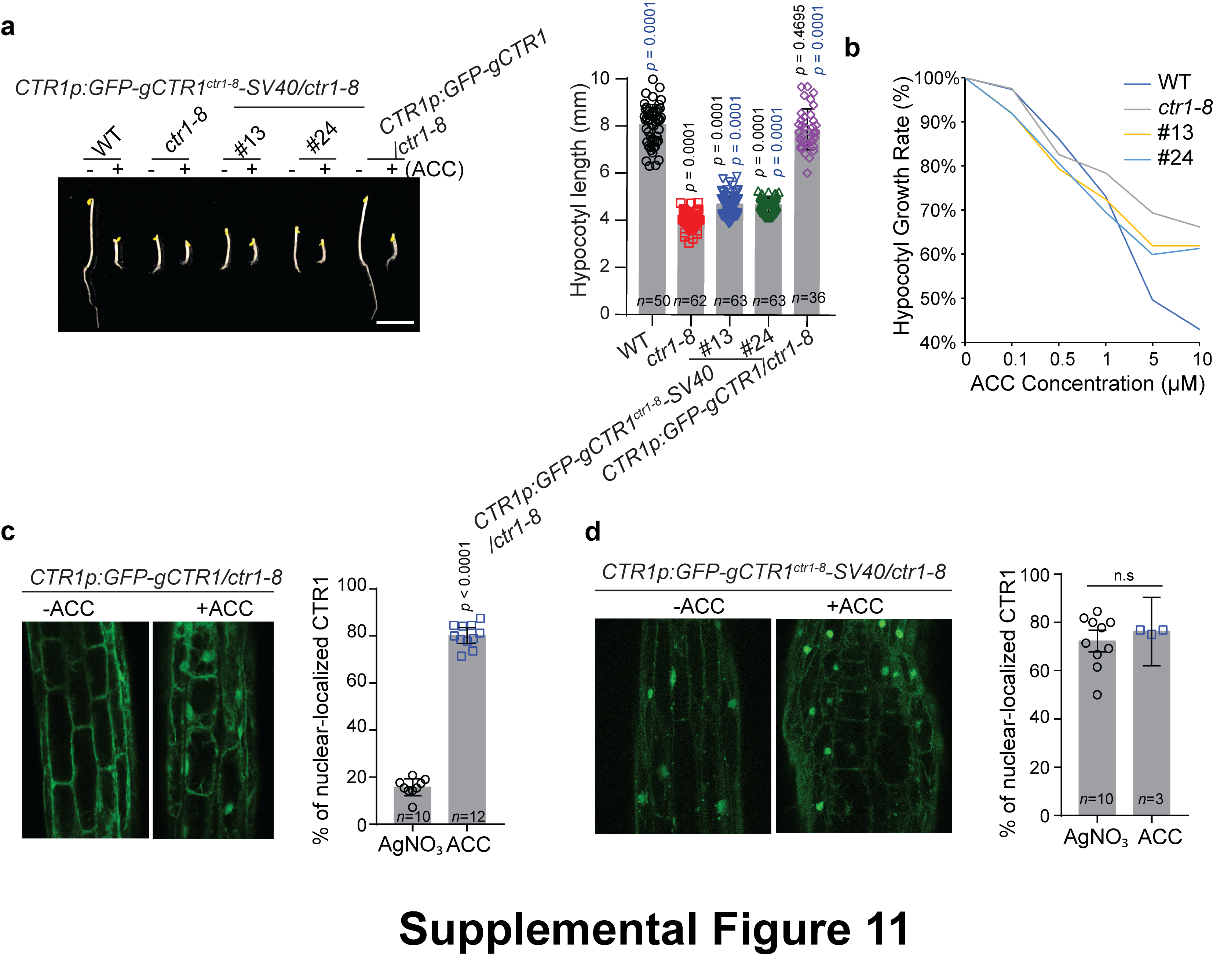
Supplementary Figure** **11. Constitutive nuclear localization of GFP-CTR1-8-SV40 NLS.**

**a**, The wild-type *GFP-CTR1* transgene, but not *GFP-gCTR1-8-SV40*, fully rescued *ctr1-8*. Seedlings were grown for 3 d in the dark with or without ACC or ethylene. The graph represents the quantification of hypocotyl lengths of seedlings grown on growth medium without 10 µM ACC. Significance was determined by one-way ANOVA, Dunnett's multiple comparisons test compared to WT (black) and *ctr1-2* (blue). Error bars, SD. Scale bar, 5 mm. **b**, The hypocotyl growth rate of *CTR1p:*GFP-CTR1^ctr1-8^-SV40/ctr1-8 seedlings in response to ACC. More than 32 seedlings per genotype were used to calculate the hypocotyl growth rate of the seedlings at different concentrations of ACC. **c-d**, Three-d-old dark-grown seedlings were treated with or without 200 μM ACC for 2 h and the localization of GFP-CTR1 was imaged. The graphs represent the ratio of nuclear-localized CTR1 in dark-grown seedlings. Error bars, SE. A two-tailed student *t-*test was used to determine statistical significance.


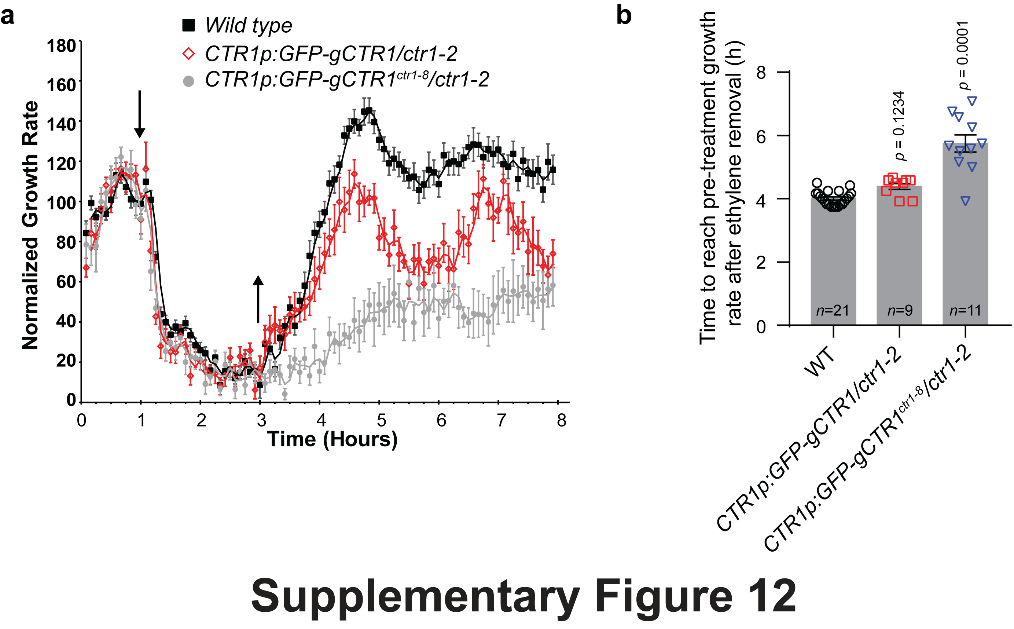


**Supplementary Figure 12. The *WT CTR1* transgene complements the *ctr1-2*, whereas the *CTR1-8* transgene only rescues ethylene response kinetics, not recovery kinetics. a,** The hypocotyl growth rate in response to ethylene was recorded for 1 h in air, followed by 2 h exposure to 10 ppm ethylene and then 5 h recovery in air. Ethylene was introduced 1 h after measurements were initiated (down arrow) and then removed 2 h later (up arrow). **b**, Quantification of time to reach the pre-treatment growth rate after ethylene treatment. Error bars, SE. *P*-value was determined by one-way ANOVA with Dunnett's multiple comparisons test compared to WT.


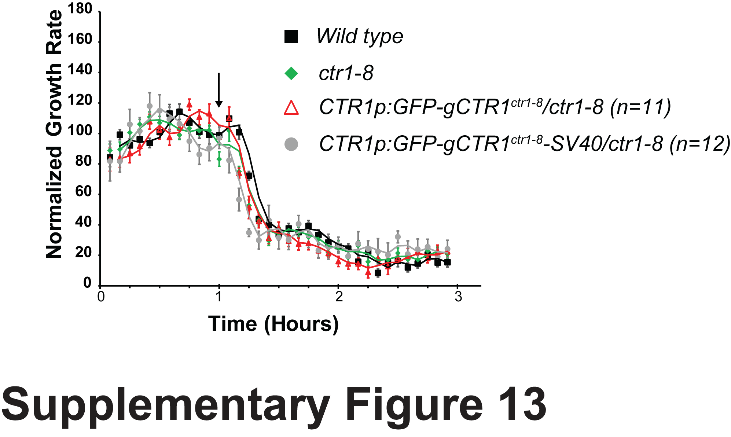


**Supplementary Figure 13. The addition of SV40 NLS to CTR1-8 does not alter the function of CTR1-8 in response to ethylene.** The hypocotyl growth rate in response to ethylene was recorded for 1 h in air, followed by 2 h exposure to 10 ppm ethylene. Ethylene was introduced 1 h after measurements were initiated (down arrow). Error bars, SE.


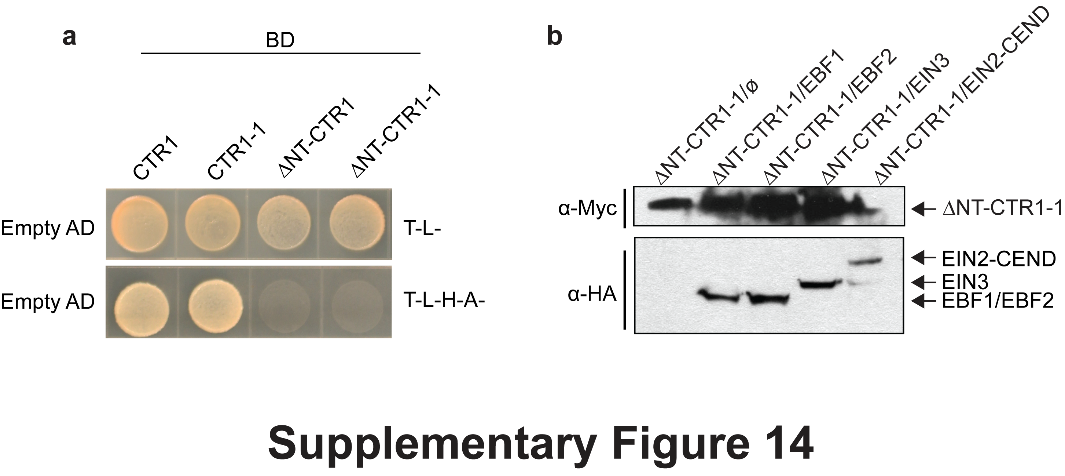


**Supplementary Figure** **14. Full-length CTR1-fused to a DNA-binding domain is autoactivated in the yeast-two-hybrid assay. a,** The coding regions of the full-length CTR1 or N-terminally deleted CTR1 with or without kinase activity were fused to a DNA-binding domain (bait). AH109 yeast strains expressing bait and empty prey plasmids were grown on selection medium. **b**, immunoblots of yeast co-expressing bait and prey proteins in Fig. 5a. Total protein extracts of yeast were analyzed by immunoblotting using anti-Myc and anti-HA antibodies. Experiments were repeated three times with similar results.


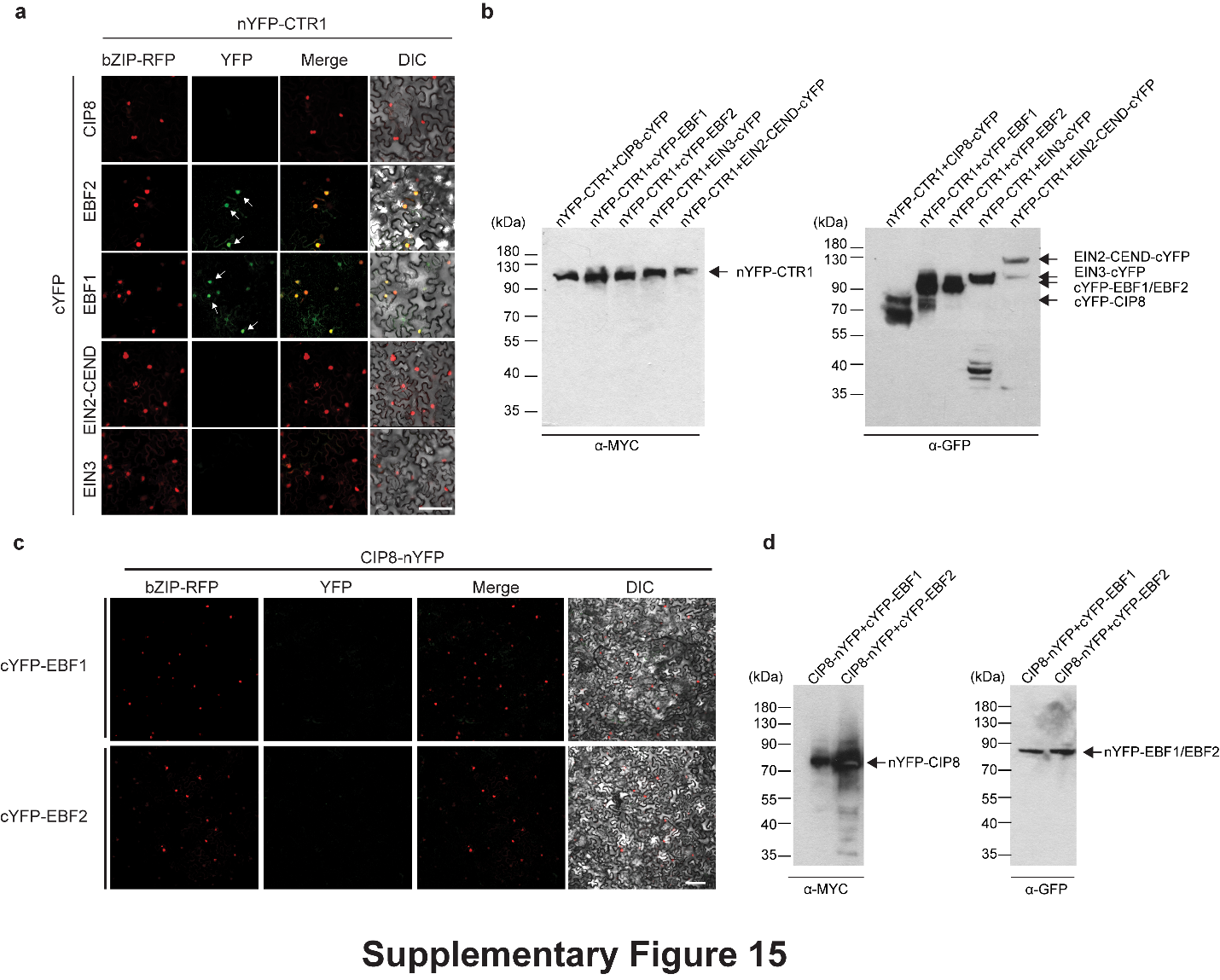


**Supplementary Figure 15. CTR1 interacts with EBF1 or EBF2 in the nucleus in the absence of ACC.** **a**, BiFC assay for full-length wild-type CTR1 and nuclear ethylene signaling proteins in *N. benthamiana* in the absence of ACC. Tobacco leaves were infiltrated with agrobacteria transformed with the indicated constructs along with the bZIP-RFP nuclear marker, and further incubated for 3 d before imaging protein-protein interactions with confocal microscopy. CIP8, COP1-interacting protein 8, was used as a negative control. Scale bar, 100 μm. **b**, Immunoblots of total protein extracts from *N. benthamiana* leaves transiently co-expressing nYFP-CTR1 and cYFP-CIP8 and control proteins in **Fig. 5b**. Anti-Myc and anti-GFP antibodies were used to detect nYFP and cYFP-fused proteins. **c**, EBF1 and EBF2 does not interact with other CIP8 control in *N. benthamiana*. **d**, immunoblots of total protein extracts of the *N. benthamiana* leaves in (**c**). All imaging and immunoblot analyses were repeated three times with same results.


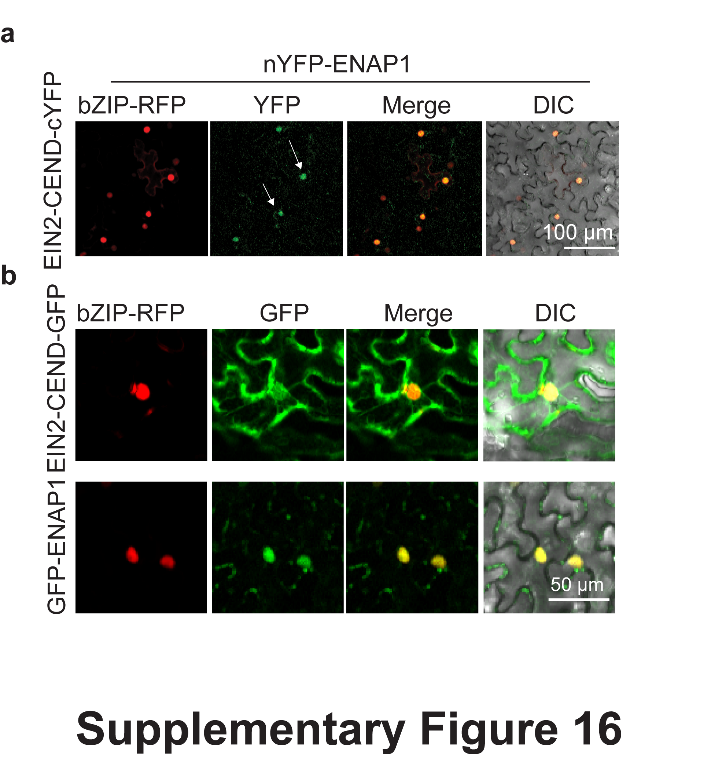


**Supplementary Figure 16. EIN2-CEND interacts with ENAP1 in the nucleus**.

**a,** *N. benthamiana* was infiltrated with agrobacteria co-transformed with EIN2-CEND-cYFP, nYFP-ENAP1, and the bZIP-RFP nuclear marker, and incubated for 3 d before imaging. Arrows indicate the reconstituted YFP signals. Scale bar, 100 μm. **b**, EIN2-CEND and ENAP2 localize in the nucleus. Scale bar, 50 μm. Three independent experiments were performed with similar results.

**
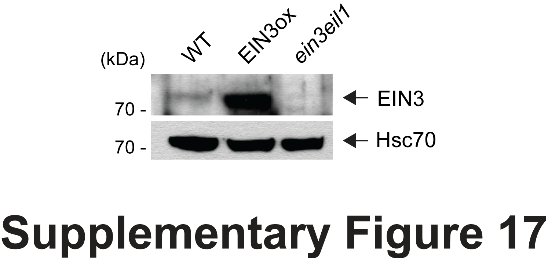
**

**Supplementary Figure 17. Confirmation of the EIN3 antibody used in the study.** Total protein extracts of etiolated WT, EIN3 overexpression (EIN3ox), and *ein3eil1* mutant without ethylene treatment were analyzed by immunoblotting using an EIN3 antibody. Three independent experiments were performed with similar results.

**
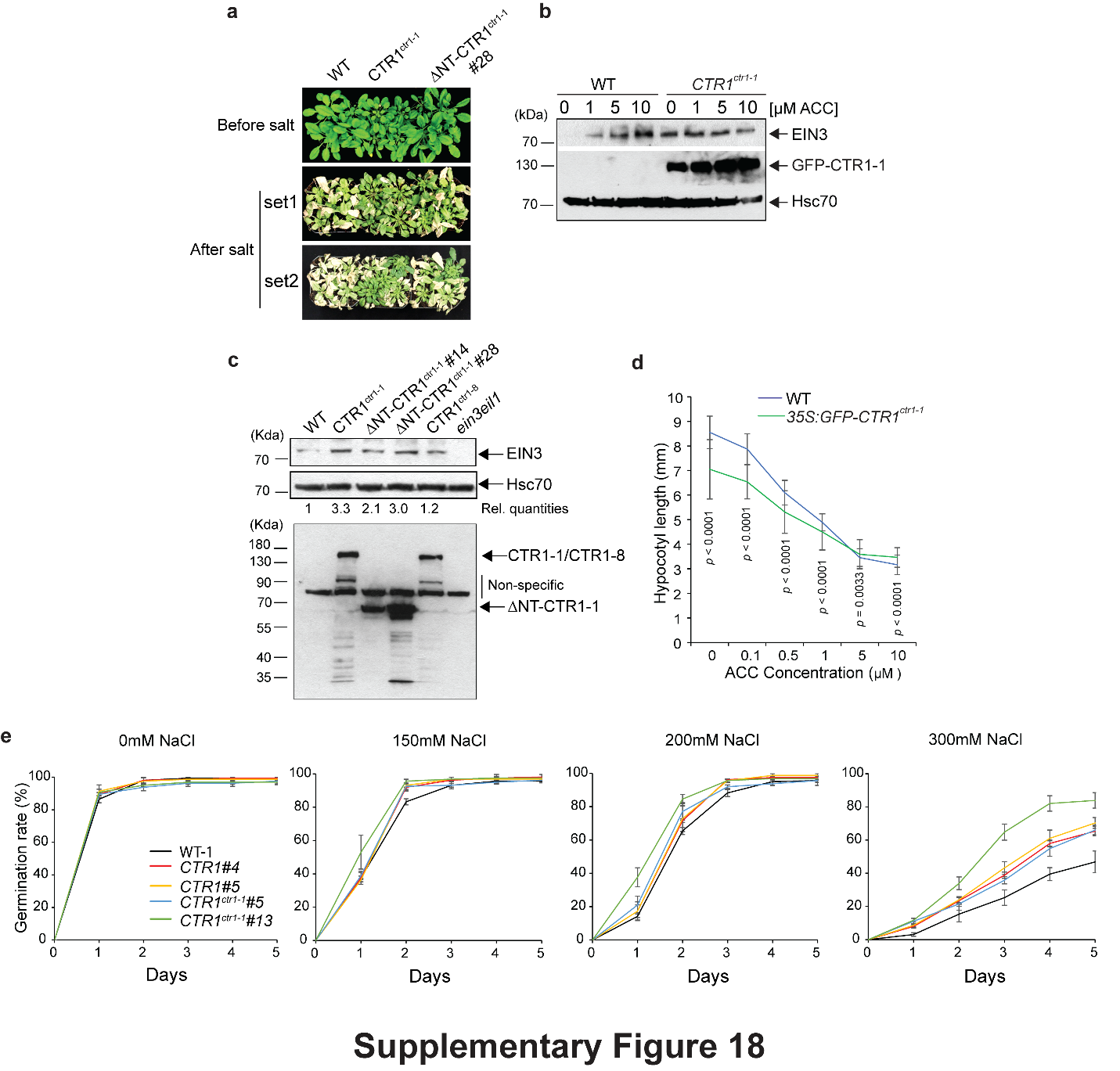
Supplementary Figure 18. The enhanced stress resilience to salinity correlates with nuclear-localized CTR1 and its associated increases in EIN3 levels. a,** Four-week-old seedlings were irrigated with 300 mM NaCl solution every 4 days for 12-d, then watered normally for 7-d. **b**, Three-day-old dark-grown *35:GFP-CTR1^ctr1-1^* seedlings were treated with the indicated concentrations of ACC for 2 h, and total protein extracts were used for immunoblotting with anti-EIN3, GFP, and Hsc70 antibodies. **c**, EIN3 and CTR1 protein levels of seedlings expressing WT CTR1 and CTR1 variants in the wild type background. Rel. quantities represent the ratio of the intensity of the EIN3 bands to Hsc70 band signals, and these values are expressed relative to the intensity of EIN3/Hsc70 in seedlings expressing WT CTR1 (lane 1) value, which was set to 1.

**d**, ACC dose-response curves for the hypocotyl length of 3-d-old dark-grown wild-type and *35:GFP-CTR1^ctr1-1^* seedlings. Error bars, SD (*n* ≥ 24 seedlings for each ACC concentration), Two-tailed student’s *t*-test. **e**. Germination analysis of seedlings on different concentrations of NaCl. Two independent lines of *35:GFP-CTR1* and *35:GFP-CTR1^ctr1-1^* seeds were germinated on media containing different concentrations of NaCl. The graphs present the germination rate of each genotype along the wildtype. Error bars, SD of three biological replicates. Each replicate contains 54 seedlings (*n*= 3 Biological replicates) per genotype.

**
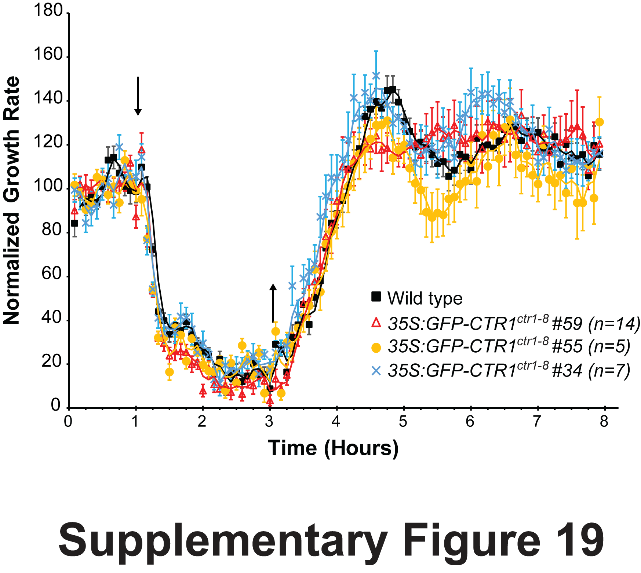
**

**Supplementary Figure 19. The overexpression of CTR1-8 protein does not influence ethylene response and recovery kinetics.** The hypocotyl growth rate in response to ethylene was recorded for 1 h in air, followed by 2 h exposure to 10 ppm ethylene and then 5 h recovery in air. Ethylene was introduced 1 h after measurements were initiated (down arrow) and then removed 2 h later (up arrow). Error bars, SE.

**
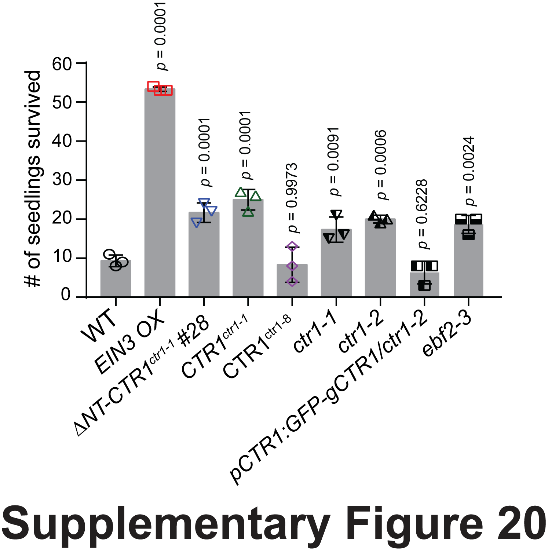
**

**Supplementary Figure 20. Ethylene signaling positively influences salinity tolerance.**

Survival rate of seedlings expressing CTR1 variants, *ctr1-1*, *ctr1-2*, *ebf2-3*, and EIN3 overexpression lines. Seedlings were grown on MS medium containing with or without 175 mM NaCl for 2-weeks and the survival rate of the seedlings were counted. A graph represents the quantification of the survival rate of seedlings. Error bars, SD for three biological replicates. Each replicate contains 54 seedlings for genotype. Statistical significance was determined using one-way ANOVA with Dunnett's multiple comparisons test, with comparisons made to the WT control group.

**
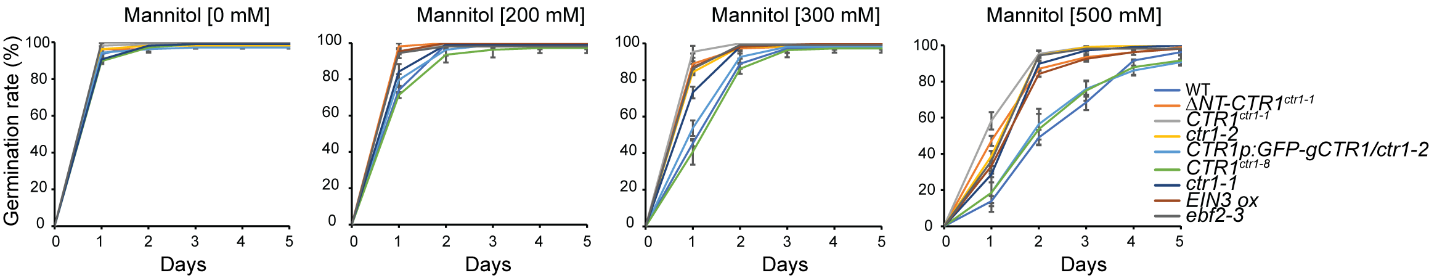
**

**Supplementary Figure 21. Germination analysis of seedlings on different concentrations of mannitol.** Arabidopsis seeds were germinated on media containing different concentrations of mannitol. The graphs present the germination rate of each genotype. Error bars, SD of three biological replicates. Each replicate contains 36 seedlings per genotype.

**Supplemental Table 1. Average growth rate of seedlings in air.**

|  | N | Average growth rate in air  (mm/h) ± SEM) | Adjusted P value to wildtype |
| --- | --- | --- | --- |
| wildtype | 21 | 0.32 ± 0.01 |  |
| *35S:GFP-ΔNT-CTR1* | 14 | 0.37 ± 0.01 | 0.4646 |
| *35S:GFP-ΔNT-CTR1 ^ctr1-1^* | 9 | 0.38 ± 0.01 | 0.3023 |
| *ctr1-8* | 47 | 0.21 ± 0.01 | <0.0001 |
| *CTR1p:GFP-CTR1 ^ctr1-8^-SV40/ctr1-8* | 22 | 0.28 ± 0.01 | 0.2593 |
| *CTR1p:GFP-CTR1/ctr1-8* | 6 | 0.29 ± 0.01 | 0.9574 |
| *35S:GFP-CTR1* | 10 | 0.29 ± 0.01 | 0.8581 |
| *35S:GFP-CTR1 ^ctr1-1^* | 6 | 0.27 ± 0.01 | 0.4297 |
| *pCTR1:GFP-CTR1/ctr1-2 #1* | 10 | 0.32 ± 0.01 | 0.9999 |
| *pCTR1:GFP-CTR1 ^ctr1-8^/ctr1-2* | 12 | 0.36 ± 0.0 | 0.6079 |

**Supplemental Table 2. Average time to reach pre-treatment growth rate after ethylene removal**

| \|  \| N \| Average time (hours) to reach pre-treatment growth rate after ethylene removal (Ave ± SEM) \| Adjusted P value to wildtype \| \| --- \| --- \| --- \| --- \| \| wildtype \| 21 \| 4.01 ± 0.05 \|  \| \| *35S:GFP-ΔNT-CTR1* \| 14 \| 4.47 ± 0.07 \| 0.0001 \| \| *35S:GFP-ΔNT-CTR1^ctr1-1^* \| 9 \| 4.52 ± 0.14 \| 0.0002 \| \| *ctr1-8* \| 47 \| 4.61 ± 0.08 \| 0.0001 \| \| *CTR1p:GFP-CTR1 ^ctr1-8^-SV40/ctr1-8* \| 22 \| 5.42 ± 0.10 \| 0.0001 \| \| *CTR1p:GFP-CTR1/ctr1-8* \| 6 \| 3.90 ± 0.09 \| 0.9111 \| \| *35S:GFP-CTR1* \| 10 \| 4.48 ± 0.09 \| 0.0001 \| \| *35S:GFP-CTR1 ^ctr1-1^* \| 6 \| 4.64 ± 0.11 \| 0.0001 \| \| *pCTR1:GFP-CTR1/ctr1-2 #1* \| 10 \| 4.39 ± 0.10 \| 0.1234 \| \| *pCTR1:GFP-CTR1 ^ctr1-8^/ctr1-2* \| 12 \| 5.75 ± 0.27 \| 0.0001 \|   **Supplemental Table 3. Primers used in this study**   \| **Primers for ENTRY gateway vectors** \| \| \| --- \| --- \| \| CTR1-CACC-F \| CACCATGGAAATGCCCGGTAGAAGATCT \| \| CTR1-R-WS \| TTACAAATCCGAGCGGTTGGGC \| \| ∆NT-CTR1-CACC-F \| CACCATGAACAGGGCAAATAGGGAACTTGG \| \| ∆NT-CTR1-R-WS \| TTACAAATCCGAGCGGTTGGGC \| \| ENAP1-F \| GTCGACGGTATCGATATGGAGACGACGCCGGAGA \| \| ENAP1-R \| GGCGGCCGCTCTAGACAACTTCTTACCTGAAGCAGCTC \| \| CIP8-F \| GGGCCCCCCCTCGAGATGTCCGATGCTCCGTCGTCTTC \| \| CIP8-R \| CGCTCTAGAACTAGTGTAACGAGAAGTTGAAGAAGAAGAAG \| \| **Mutagenesis primers for CTR1** \| \| \| ctr1-1-D694E-F \| CAGTCAAGGTTTGTGAGTTTGGTCTCTCGCGAT \| \| ctr1-1-D694E-R \| ATCGCGAGAGACCAAACTCACAAACCTTGACTG \| \| ctr1-8-G354E-F \| GTAGCCTCTCTGTTGAACTCTGCAGACATCG \| \| ctr1-8-G354E-R \| CGATGTCTGCAGAGTTCAACAGAGAGGCTAC \| \| ctr1-1-gD694E-F \| ATTTCAGGTTTGTGAATTTGGTCTCTCGCGA \| \| ctr1-1-gD694E-R \| TCGCGAGAGACCAAATTCACAAACCTGAAAT \| \| ctr1-8-gG354E-F \| GTAGCCTCTCTGTTGAACTCTGCAGACATCG \| \| ctr1-8-gG354E-R \| CGATGTCTGCAGAGTTCAACAGAGAGGCTAC \| \| CTR-T704A-F \| CGATTGAAGGCCAGCGCATTTCTTTCCTCGAA \| \| CTR-T704A-R \| TTCGAGGAAAGAAATGCGCTGGCCTTCAATCG \| \| CTR-T707A-F \| GCCAGCACGTTTCTTGCTTCGAAGTCAGCAGC \| \| CTR-T707A-R \| GCTGCTGACTTCGAAGCAAGAAACGTGCTGGC \| \| CTR-T710A-F \| TTTCTTTCCTCGAAGGCAGCAGCTGGAACCCC \| \| CTR-T710A-R \| GGGGTTCCAGCTGCTGCCTTCGAGGAAAGAAA \| \| **Primers for native promoter-driven CTR1 transgenic plants** \| \| \| Promoter CTR1-F \| TGGTTAGAGAGGCCTGGAGAAGTAGAAAAGAAAACGAAAA \| \| Promoter CTR1-R \| CTCCTCGCCCTTGCCCATGGAAAAGAGAGTTATCGCGAT \| \| Genomic CTR1-F \| GGACGAGCTGTACAAGATGGAAATGCCCGGTAGAAGA \| \| Genomic CTR1-R \| AATTAACTCTCTAGATTACAAATCCGAGCGGTTGG \| \| Genomic ∆NT-CTR1-F \| GGACGAGCTGTACAAGAACAGGGCAAATAGGGAACTTGGA \| \| Genomic ∆NT-CTR1-R \| AATTAACTCTCTAGATTACAAATCCGAGCGGTTGG \| \| YFP-F \| GCGATAACTCTCTTTTCCATGGGCAAGGGCGAGGAGCTG \| \| YFP-R \| TCTACCGGGCATTTCCATCTTGTACAGCTCGTCCATGC \| \| GFP-F \| GCGATAACTCTCTTTTCCATGGTGAGCAAGGGCG \| \| GFP-R \| TCTACCGGGCATTTCCATCTTGTACAGCTCGTCCATGC \| \| CTR1-SV40-NLS-F \| TAGATGAGAGACGTCGCCTGAGTATG \| \| SV40-NLS-R1 \| CTTCTTCTTAGGCAAATCCGAGCGGTTGGGCG \| \| SV40-NLS-R2 \| TTAAACCTTTCTCTTCTTCTTAGGCAAATCCGAGC \| \| SV40-NLS-R3 \| AATTAACTCTCTAGATTAAACCTTTCTCTTCTTCTTAGGCA \| \| Promoter EIN2-F \| CGCTCTAGAACTAGTACCCAATGATCCGTACGCAGTC \| \| Promoter EIN2-R \| GCTGAGGTCTCACATTCACAATTTC \| \| Genomic EIN2-F1 \| GAAATTGTGAATGTGAGACCTCAGC \| \| Genomic EIN2-R1 \| TCTTTCCTCTGGAGGGCTCGAAC \| \| Genomic EIN2-F2 \| GTTCGAGCCCTCCAGAGGAAAGA \| \| Genomic EIN2-R2 \| GACTGCGTACGGATCATTGGGTATGGTGAGCAAG \| \| mCherry-F \| ATCATTGGGTATGGTGAGCAAGGGCGAGGAG \| \| mCherry-R \| ATCGGGGATCGGATCCCTACTTGTACAGCTCGTCCATG \| \| **Primers for yeast-2-hybrid constructs** \| \| \| ∆NT-CTR1-BD-F \| AGGAGGACCTGCATATGAACAGGGCAAATAGGGAAC \| \| ∆NT-CTR1-BD-R \| TAGTTATGCGGCCGCTGCAGTTACAAATCCGAGCGGTTGGG \| \| ∆NT-CTR1-1-BD-F \| AGGAGGACCTGCATATGAACAGGGCAAATAGGGAAC \| \| ∆NT-CTR1-1-BD-R \| TAGTTATGCGGCCGCTGCAGTTACAAATCCGAGCGGTTGGG \| \| CTR1-FL-BD-F \| AGGAGGACCTGCATATGATGGAAATGCCCGGTAGAAG \| \| CTR1-FL-BD-R \| TAGTTATGCGGCCGCTGCAGTTACAAATCCGAGCGGTTGGG \| \| CTR1-1-BD-F \| AGGAGGACCTGCATATGATGGAAATGCCCGGTAGAAG \| \| CTR1-1-BD-R \| TAGTTATGCGGCCGCTGCAGTTACAAATCCGAGCGGTTGGG \| \| EBF1-AD-F \| CAGATTACGCTCATATGATGTCTCAGATCTTTAGTTTTGCCG \| \| EBF1-AD-R \| ATGGATCCCGTATCGATTCAGGAGAGGATGTCACATTTG \| \| EBF2-AD-F \| CAGATTACGCTCATATGATGTCTGGAATCTTCAGATTTAGTG \| \| EBF2-AD-R \| ATGGATCCCGTATCGATTTAGTAGAGTATATCGCACCTCC \| \| EIN2-CEND-AD-F \| CAGATTACGCTCATATGACGCCGCTGAAATCTGCG \| \| EIN2-CEND-AD-R \| ATGGATCCCGTATCGATTCAACCCAATGATCCGTACGC \| \| EIN3-AD-F \| GGAGGCCAGTGAATTCATGATGTTTAATGAGATGGGAATG \| \| EIN3-AD-R \| TCATCTGCAGCTCGAGTTAGAACCATATGGATACATCTTGC \| \| **Primers for quantitative RT-PCR** \| \| \| ERF1-RT-F \| ACGTTCTCAACCGCCTACAG \| \| ERF1-RT-R \| CGGACTCGCTCTCTGGTG \| \| RD29A-RT-F \| GTGGGCTTTGGTGACGAGTC \| \| RD29A-RT-R \| GTGTCCATTCCAGTTTCAGTCTTC \| \| DREB2A-RT-F \| GACCTAAATGGCGACGATGT \| \| DREB2A-RT-R \| TCGAGCTGAAACGGAGGTAT \| \| COR47-RT-F \| AGCATTCTTGGCGCTTATTGCTCG \| \| COR47-RT-R  ACTIN2-RT-F  ACTIN2-RT-R \| TCCCTGCTCGTGAGAAATGTTGGA  ATTCAGATGCCCAGAAGTCTTGTT  ACGGTCAGCGATACCTGAGAAC \| |
| --- | --- | --- | --- | --- | --- | --- | --- | --- | --- | --- | --- | --- | --- | --- | --- | --- | --- | --- | --- | --- | --- | --- | --- | --- | --- | --- | --- | --- | --- | --- | --- | --- | --- | --- | --- | --- | --- | --- | --- | --- | --- | --- | --- | --- | --- | --- | --- | --- | --- | --- | --- | --- | --- | --- | --- | --- | --- | --- | --- | --- | --- | --- | --- | --- | --- | --- | --- | --- | --- | --- | --- | --- | --- | --- | --- | --- | --- | --- | --- | --- | --- | --- | --- | --- | --- | --- | --- | --- | --- | --- | --- | --- | --- | --- | --- | --- | --- | --- | --- | --- | --- | --- | --- | --- | --- | --- | --- | --- | --- | --- | --- | --- | --- | --- | --- | --- | --- | --- | --- | --- | --- | --- | --- | --- | --- | --- | --- | --- | --- | --- | --- | --- | --- | --- | --- | --- | --- | --- | --- | --- | --- | --- | --- | --- | --- | --- | --- | --- | --- | --- | --- | --- | --- | --- | --- | --- | --- | --- | --- | --- | --- | --- | --- | --- | --- | --- | --- | --- | --- | --- | --- | --- | --- | --- | --- | --- | --- | --- | --- | --- | --- | --- | --- | --- | --- | --- | --- | --- | --- | --- |
