# Supplementary material for: Ethylene-triggered subcellular trafficking of CTR1 enhances the response to ethylene gas
Source: Nat Commun. 2023 Jan 23;14:365. doi: 10.1038/s41467-023-35975-6 (PMC9870993; doi:10.1038/s41467-023-35975-6)
Supplement: Supplementary file 3 — Reporting Summary [file 41467_2023_35975_MOESM3_ESM.pdf]

nature portfolio

Corresponding author(s): Gyeong Mee Yoon

Last updated by author(s): 1/10/2023



# Reporting Summary

Nature Portfolio wishes to improve the reproducibility of the work that we publish. This form provides structure for consistency and transparency in reporting. For further information on Nature Portfolio policies, see our Editorial Policies and the Editorial Policy Checklist.

## Statistics

For all statistical analyses, confirm that the following items are present in the figure legend, table legend, main text, or Methods section.

| n/a | Confirmed | |
|---|---|---|
| ☐ | ☒ | The exact sample size (*n*) for each experimental group/condition, given as a discrete number and unit of measurement |
| ☐ | ☒ | A statement on whether measurements were taken from distinct samples or whether the same sample was measured repeatedly |
| ☐ | ☒ | The statistical test(s) used AND whether they are one- or two-sided<br>*Only common tests should be described solely by name; describe more complex techniques in the Methods section.* |
| ☒ | ☐ | A description of all covariates tested |
| ☒ | ☐ | A description of any assumptions or corrections, such as tests of normality and adjustment for multiple comparisons |
| ☒ | ☐ | A full description of the statistical parameters including central tendency (e.g. means) or other basic estimates (e.g. regression coefficient) AND variation (e.g. standard deviation) or associated estimates of uncertainty (e.g. confidence intervals) |
| ☐ | ☒ | For null hypothesis testing, the test statistic (e.g. *F*, *t*, *r*) with confidence intervals, effect sizes, degrees of freedom and *P* value noted<br>*Give P values as exact values whenever suitable.* |
| ☒ | ☐ | For Bayesian analysis, information on the choice of priors and Markov chain Monte Carlo settings |
| ☒ | ☐ | For hierarchical and complex designs, identification of the appropriate level for tests and full reporting of outcomes |
| ☒ | ☐ | Estimates of effect sizes (e.g. Cohen's *d*, Pearson's *r*), indicating how they were calculated |

*Our web collection on statistics for biologists contains articles on many of the points above.*

## Software and code

Policy information about availability of computer code

| | |
|---|---|
| Data collection | Microscope images are collected from Zeiss 880 Upright Confocal |
| Data analysis | The band intensities of western blotting was calculated using Image J software (ImageJ bundled with 64-bit Java 1.8.0_172) |

For manuscripts utilizing custom algorithms or software that are central to the research but not yet described in published literature, software must be made available to editors and reviewers. We strongly encourage code deposition in a community repository (e.g. GitHub). See the Nature Portfolio guidelines for submitting code & software for further information.

## Data

Policy information about availability of data

All manuscripts must include a data availability statement. This statement should provide the following information, where applicable:

- Accession codes, unique identifiers, or web links for publicly available datasets
- A description of any restrictions on data availability
- For clinical datasets or third party data, please ensure that the statement adheres to our policy

All data are available in the manuscript including supplemental data or through contacting the corresponding authors.



# Field-specific reporting

# Life sciences study design

All studies must disclose on these points even when the disclosure is negative.

| | |
|---|---|
| Sample size | The sample size for each experiment was not determined using statistical methods, but it was sufficient to achieve statistical significance and reproducibility. Each replicate was also a biologically independent sample. In confocal imaging experiments, we used at least three independent T2 lines to verify subcellular localization, and then used at least two independent lines, each with a minimum of 10 seedlings, for further imaging. For measuring hypocotyl length, we followed established standards in the literature and used at least 19 seedlings per genotype. |
| Data exclusions | No data were excluded |
| Replication | All experimental results were successfully replicated. To ensure accuracy, we performed three biological replicates for qRT-PCR experiments. In addition, we used multiple biological samples and at least three independent lines for all confocal imaging experiments to confirm subcellular localization. Similarly, we repeated BiFC, Y2H, and co-IP experiments at least three times. We also performed time-lapse growth kinetics experiments multiple times, using at least six biological replicates for each genotype. |
| Randomization | In all imaging experiments, randomization was not applied, as each genotype was treated and analyzed separately. For the ACC treatment and control groups, the seedlings were grown on the same growth medium and under identical growth conditions. After three days of growth, the seedlings were randomly divided into two groups for treatment with ACC or a vehicle solution. The plants used for the analysis of each genotype were also selected randomly. |
| Blinding | In this study, no blinding was implemented as all genotypes were treated separately with chemicals and their response to the treatments was directly observed for changes in movement or ACC responses. |

# Reporting for specific materials, systems and methods

We require information from authors about some types of materials, experimental systems and methods used in many studies. Here, indicate whether each material, system or method listed is relevant to your study. If you are not sure if a list item applies to your research, read the appropriate section before selecting a response.

## Materials & experimental systems

| n/a | Involved in the study | |
|---|---|---|
| ☐ | ☒ | Antibodies |
| ☒ | ☐ | Eukaryotic cell lines |
| ☒ | ☐ | Palaeontology and archaeology |
| ☒ | ☐ | Animals and other organisms |
| ☒ | ☐ | Human research participants |
| ☒ | ☐ | Clinical data |
| ☒ | ☐ | Dual use research of concern |

## Methods

| n/a | Involved in the study | |
|---|---|---|
| ☒ | ☐ | ChIP-seq |
| ☒ | ☐ | Flow cytometry |
| ☒ | ☐ | MRI-based neuroimaging |

## Antibodies

| | |
|---|---|
| Antibodies used | anti-GFP(Sigma-Adriatic, #Cat 11814460001); anti-HSC70 (Enzolifesceinces, #Cat ADI-SPA-818-F); anti-Histon H3 (Novusbio, #Cat NB500-171); anti-EIN3 (Agrisera, #Cat AS194273); anti-CTR1 (in-house); Goat anti-rat-HRP (Santa Cruz, Cat#sc2006); Goat anti-mouse-HRP (Invitrogen, #Cat31430); anti-BIP (Santa Cruz, Cat# sc-8017); anti-Myc (Sigma, Cat# 11814150001);anti-HA (Roche, cat#11867423001). |
| Validation | All antibodies used in this study were validated by the supplier and previous publications. |
